# Peripheral T Cell Development and Immunophenotyping of Twins with Heterozygous *FOXN1* Mutations

**DOI:** 10.4049/immunohorizons.2400006

**Published:** 2024-07-15

**Authors:** Kelsey Voss, Todd Bartkowiak, Allison E. Sewell, Channing Chi, Madelyn D. Landis, Samuel Schaefer, Heather H. Pua, James A. Connelly, Jonathan M. Irish, Jeffrey C. Rathmell, Saara Kaviany

**Affiliations:** * Department of Pathology, Microbiology and Immunology, Vanderbilt University Medical Center, Nashville, TN; † Department of Cell and Developmental Biology, Vanderbilt University, Nashville, TN; ‡ Vanderbilt Center for Immunobiology, Vanderbilt University Medical Center, Nashville, TN; § Human Immunology Discovery Initiative of the Vanderbilt Center for Immunobiology, Vanderbilt University Medical Center, Nashville, TN; ¶ Department of Pediatrics, Vanderbilt University Medical Center, Nashville, TN

## Abstract

The transcription factor *FOXN1* plays an established role in thymic epithelial development to mediate selection of maturing thymocytes. Patients with heterozygous loss-of-function *FOXN1* variants are associated with T cell lymphopenia at birth and low TCR excision circles that can ultimately recover. Although CD4^+^ T cell reconstitution in these patients is not completely understood, a lower proportion of naive T cells in adults has suggested a role for homeostatic proliferation. In this study, we present an immunophenotyping study of fraternal twins with low TCR excision circles at birth. Targeted primary immunodeficiency testing revealed a heterozygous variant of uncertain significance in *FOXN1* (c.1205del, p.Pro402Leufs*148). We present the immune phenotypes of these two patients, as well as their father who carries the same *FOXN1* variant, to demonstrate an evolving immune environment over time. While *FOXN1* haploinsufficiency may contribute to thymic defects and T cell lymphopenia, we characterized the transcriptional activity and DNA binding of the heterozygous *FOXN1* variant in 293T cells and found the *FOXN1* variant to have different effects across several target genes. These data suggest multiple mechanisms for similar *FOXN1* variants pathogenicity that may be mutation specific. Increased understanding of how these variants drive transcriptional regulation to impact immune cell populations will guide the potential need for therapeutics, risk for infection or autoimmunity over time, and help inform clinical decisions for other variants that might arise.

## Introduction

The thymus controls the development of naive T cells and programs their repertoire to differentiate between “self” and “non-self.” A major component of the thymic stroma are thymic epithelial cells (TECs), which are critical players in the maturation and selection process of T cells (1). FOXN1 is a transcription factor required for TEC differentiation and function in the fetal and adult thymus (2, 3). Indeed, autosomal recessive loss-of-function mutations of *FOXN1* in humans is known to result in severe combined immunodeficiency, where patients exhibit athymia, congenital alopecia, and nail dystrophy (4, 5). Heterozygous loss-of-function *FOXN1* variants are associated with low TCR excision circles (TRECs) and T cell lymphopenia at birth (6, 7). However, the T cell lymphopenia in heterozygous patients evolves over time and can resolve. As these patients are followed into adulthood, they exhibit a lower proportion of naive T cells compared with early in life, suggesting that likely homeostatic proliferation is promoting CD4 T cell reconstitution over time (6). Nonetheless, how specific *FOXN1* mutations can impact T cell development in different stages of life is still unclear.

Immunodeficiencies caused by heterozygous FOXN1 mutations have mostly been described as causing pathology by reduced gene dosage of FOXN1 or haploinsufficiency (6, 8). However, one recent report also described a C-terminal mutant of FOXN1 that exerts a proposed dominant negative effect on wild-type (WT) FOXN1 (9). Therefore, heterozygous mutations could result in either haploinsufficiency or dominant-negative pathologies. In this study, we present a case of fraternal twins with T cell lymphopenia at birth, with quantitatively normal B and NK cell populations, consistent with T-B^+^NK^+^ severe combined immunodeficiency. Targeted primary immunodeficiency testing revealed a *FOXN1* heterozygous variant of uncertain significance (VUS): c.1205del, p.Pro402Leufs*148. We conducted comprehensive immunophenotyping of the twins and their father, a carrier of the same *FOXN1* variant, and confirmed that the c.1205del heterozygous variant is pathogenic. Furthermore, we tested the c.1205del variant for its ability to activate known target genes and DNA binding to their promoters, revealing a complex picture that cannot be explained by gene dosage effects alone. Thus, the c.1205del *FOXN1* variant is capable of some dominant-negative activity in vitro, but specific to only certain *FOXN1* targets. These findings highlight the potential complexity of *FOXN1* heterozygous variants and the need for an improved understanding of their actions on gene targets involved in T cell development.

## Materials and Methods

### Study approval

Written informed consent was obtained from all participants prior to participation, and this study was conducted in accordance with the Declaration of Helsinki, approved by the Institutional Review Board (IRB) at Vanderbilt University Medical Center.

### Donor PBMC collection and processing

PBMCs were collected from healthy volunteers with written informed consent under IRB protocols 131311 and 191562. The patients with *FOXN1* heterozygous mutations were collected with written informed consent under IRB protocol 182228. Samples were deidentified prior to processing, and no other information was obtained from healthy individuals outside of a brief health survey. The healthy donor controls were adults and not age-matched pediatric patients. IRB consent for healthy donors does not allow for further identifiers. Blood was collected by venipuncture into heparinized collection tubes (Becton Dickinson; 100 ml/donor). Whole blood was diluted 1:4 with PBS before being overlaid onto a Ficoll-Paque Plus density gradient (GE Life Sciences). Blood was then centrifuged at 400 × *g* for 30 min without braking. Buffy coats were isolated, washed with 1× PBS, and centrifuged at 500 × *g* for 10 min. Cell pellets were then resuspended in ACK (ammonium-chloride-potassium) lysis buffer for 5 min, washed in 1× PBS, and cryopreserved at 1 × 10^7^ cells/ml at −80°C in 10% DMSO in FBS.

### Mass cytometry (cytometry by time-of-flight)

Cryopreserved samples were rapidly thawed in a 37°C water bath and resuspended in complete RPMI supplemented with 10% FBS and 50 U/ml of penicillin–streptomycin (Thermo Scientific HyClone). Cell suspensions were processed and stained as previously described (10–12). Briefly, cells were washed once with serum free RPMI and subsequently stained with ^103^Rh Cell-ID Intercalator (Fluidigm) at a final concentration of 1 µM for 5 min at room temperature. Staining was quenched with complete RPMI before washing with PBS 1% BSA. Cells were resuspended in PBS/BSA and added to the appropriate Ab mixture of cell-surface staining Abs as previously described (13) and incubated at room temperature for 30 min. Samples were washed in 1% PBS/BSA before fixation in 2% paraformaldehyde for 10 min at room temperature. Cells were again washed in PBS and fixed in ice cold methanol with gentle vortexing before storage at −20°C. On the day of data collection, samples stored at −20°C were washed in PBS/BSA and resuspended in an Ab mixture of intracellular stains for 30 min. Iridium Cell-ID Intercalator was added at a final concentration of 125 nM and incubated at room temperature for at least 30 min. Cells were then washed and resuspended in ultrapure deionized water, mixed with 10% EQ Four Element Calibration Beads (Fluidigm) and filtered through a 40 µM FACS filter tube before data collection. Data were collected on a Helios CyTOF (cytometry by time-of-flight) 3.0 (Fluidigm). Quality control and tuning processes were performed following the guidelines for the daily instrument operation. Data were collected as FCS files.

### Data preprocessing

Raw mass cytometry files were normalized using the MATLAB bead normalization tool (14). Files were then uploaded to the cloud-based analysis platform Cytobank. Before automated high-dimensional data analysis, the mass cytometry data were transformed with a cofactor of 5 using an inverse hyperbolic sine (arcsinh) function. Cell doublets were first excluded using Gaussian parameters (center, offset, width, residual) as reported (10). Intact cells were gated based on DNA content (^191^Ir and ^193^Ir). Dead cells were excluded based on rhodium intercalation. Immune subsets were then manually gated using biaxial gating strategies.

### Dimensionality reduction

Analysis by t-distributed stochastic neighbor embedding (t-SNE) was performed using the Cytobank platform. For each respective panel, cells of interest (CD45^+^CD3^+^ T cells, CD45^+^CD19^+^ B cells, or CD45^+^CD3^−^CD19^−^CD56^−^ myeloid cells) were pregated before analysis. Dimensionality reduction was performed using all markers within each panel. Metabolic and phospho-protein markers were excluded to assess individual marker expression. Each t-SNE map was generated using a perplexity of 30, theta of 0.5, and 10,000 iterations.

### Statistical analysis

All CyTOF statistical analysis was performed in Cytobank, R version 3.6.1, or GraphPad Prism version 8.4.3. Where appropriate, median intensity values were transformed using the arcsinh scale normalized to the minimum intensity value across all markers. Patient values outside of the range of five pooled healthy donor samples were considered significant. Where indicated, two-way ANOVA with a Tukey correction was performed to compared differences between multiple groups. A *p* value <0.05 was considered statistically significant.

### FOXN1 expression

Custom plasmids were generated from the pCMV6-entry mammalian expression vector (OriGene, catalog no. PS100001) with either WT Foxn1 or c.1205del Foxn1 inserts. HEK293T cells (American Type Culture Collection, CRL-3126) were maintained in DMEM + 10% heat-inactivated FBS and 1% penicillin/streptomycin. For transfections, 500,000 cells/well were seeded into tissue culture–treated 12-well plates in 1.5 ml of antibiotic-free DMEM + 10% FBS. Twenty-four hours later, cells were transfected with 1 µg of WT Foxn1 or c.1205del Foxn1 plasmid DNA using the jetPRIME Polyplus-transfection reagent (VWR) as directed. Media were changed after 12 to 24 h and cells were harvested for downstream analysis at 48 h posttransfection. All quantitative PCR and chromatin immunoprecipitation (ChIP) experiments were conducted with a 2:1 ratio of WT to c.1205del Foxn1 plasmid to normalize protein expression levels.

### Immunoblot analysis

Cells were lysed and lysate was separated by SDS-PAGE as previously described (15). Protein was transferred to nitrocellulose membranes using the Transblot Turbo system (Bio-Rad). Nitrocellulose membranes were probed with either anti-FLAG (OriGene, clone OTI4C5, 1:1000), anti-Foxn1 (Invitrogen, catalog no. PA5-21618, 1:1000) or β-actin (Cell Signaling Technologies, catalog no. 3700S, 1:5000) and imaged using the Odyssey CLx instrument (LI-COR). Blots were incubated with IRDye anti-rabbit or anti-mouse secondary Abs (LI-COR) at 1:10,000, and band intensity quantification was conducted with Studio Lite software (LI-COR).

### Quantitative RT-PCR

RNA was isolated from 293T cells following the Qiagen Mini RNeasy kit with DNase digestion.

RNA (300 ng) was used for cDNA conversion following the Bio-Rad iScript cDNA synthesis kit. Quantitative RT-PCR was performed on the Bio-Rad CFX96 thermocycler machine and SsoAdvanced Universal SYBR Green supermix with the following program: 95°C for 30 s denaturation, 95°C for 15 s, and 60°C for 30 s for 39 cycles, then 95°C for 5 s followed by melt curve analysis. Primer sequences were as follows: PSMB11 forward, 5′-GTGGGCTCTGGATCTCCCTA-3′, reverse, 5′-GAGCCCCCTGAATAGGCATC-3′; PSMB9 forward, 5′-CAAGCGGGGGTGTCATCTAC-3′, reverse, 5′-ACCATACCAGGTTTTGGCCC-3′; CD83 forward, 5′-CACTTGTAAGTTTGCACGGCT-3′, reverse, 5′-GTAACAGACAGGCACACCCC-3′.

### ChIP

For transfections, 700,000 cells/well were seeded into tissue-culture treated six-well plates in 3 ml of antibiotic-free DMEM + 10% FBS. ChIP was performed using the Simple ChIP Plus enzymatic ChIP kit (Cell Signaling Technology) with the addition of either 5 µg of anti-FLAG (OriGene, clone OTI4C5), 5 µg of IgG2a control, or 2 μg of anti-histone H3 (Cell Signaling Technology, catalog no. 4620) Abs for immunoprecipitation. The primer sequences for the target promoter regions were as follows: PSMB11 forward, 5′-AGCCAATCCCAAAGCCTGAA-3′, reverse, 5′-GGGATAGGAGGGACGCTACA-3′; CD83 forward, 5′-TCCTGAGCTGCGGTAGGG-3′, reverse, 5′-AGGCTACAAGAAAGAGAGAGCG-3′. Control RPL30 primers were provided in the Cell Signaling Technology kit. Cycling conditions were as follows: 95°C for 3 min, then 40 cycles of 95°C for 15 s and 60°C for 60 s.

## Results

### Patient characteristics

The patients in this study were enrolled in the Human Immune Disease Initiative at Vanderbilt University, combining multidimensional cytometry and genomics to gain deeper insight into inborn errors of immunity. The fraternal twins presented with severely low TRECs and T cell lymphopenia at the time of newborn screening (Table I). Early bloodwork of the twins showed low numbers of CD3, CD4, and CD8^+^ T cells indicative of reduced thymic output (Supplemental Fig. 1A**–**C). Targeted genetic testing with the Invitae primary immunodeficiency panel revealed two heterozygous VUS, that is, in *DOCK8* (C.277G>T, p.Val93Leu) and *FOXN1* (c.1205del, p.Pro402Leufs*148). However, the *FOXN1* variant contains a frameshift mutation that had been recently reported as pathogenic in other patients (P14) (6). This premature deletion could be predicted to affect the transactivation domain necessary for increasing DNA affinity in a DNA binding assay (16). In this published case series, CD4^+^ T cell lymphopenia was less pronounced beyond 2 y of age, while CD8^+^ counts remained below the normal range in most infants. The twins’ lymphopenia remained significantly below the lower limit of the normal range throughout the study until the last blood collection at 23 mo of age. Of note, live attenuated vaccines were held throughout the course of investigation. At this latest time point, 40% of CD4^+^ T cells were naive and >80% of CD8^+^ T cells were naive (Supplemental Fig. 1D, 1E). Adult carriers of this previously described variant did not normalize their CD8^+^ counts and 60% had abnormal nails. In this study, the twins’ father exhibited nail dystrophy and was then found to be affected by the same variant (Supplemental Fig. 1F). He did report being ill more frequently as a child. Therefore, further immunophenotyping was performed with the hypothesis that the c.1205del *FOXN1* variant was pathogenic.

**Table I.** Laboratory results of twins <1 mo of age

Laboratory Test	Normal Value (baby)	Baby Boy	Baby Girl	Dad
Total T cell counts	1484–5327 cells/µl	86 cells/µl	514 cells/µl	1176 cells/µl
Naive T cell counts	64–95% CD4, 80–99% CD8	63% CD4, 99% CD8	78% CD4, 97% CD8	22% CD4, 48% CD8
TRECs	≥6794 copies per 10^6^ T cells	Below limit of detection	4081 copies per 10^6^ T cells	Not done
T cell proliferation (PHA)	Normal proliferation	68% of normal	Normal proliferation	Normal proliferation
Origin of T cells	Baby	Baby	Baby	Not done
Genetic testing	Normal genetic sequencing	VUS in *DOCK8* and *FOXN1*	VUS in *DOCK8* and *FOXN1*	VUS in *FOXN1*

### Immunophenotyping of FOXN1 heterozygous patients

**FIGURE 1.**
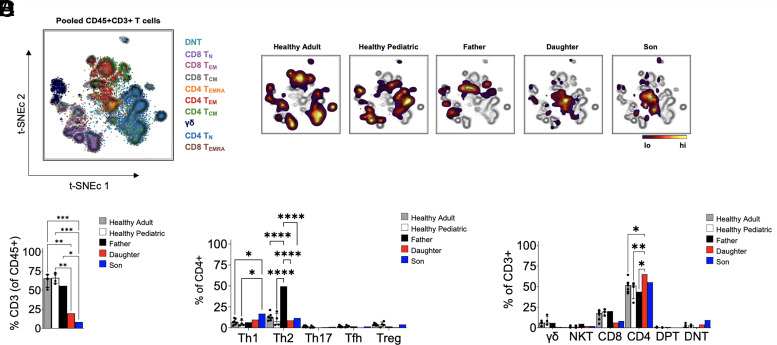
Immunophenotyping of T cell compartments by CyTOF. (**A**) Left, t-SNE analyses of pooled T cells from all individuals. Colored legend corresponds to each T cell subset on the t-SNE map. Right, Density overlays from pooled healthy adults (*n* = 7), healthy pediatric donors (*n* = 4), *FOXN1* father, daughter, and son, respectively, overlaid onto the pooled aggregates indicating each person’s T cell populations. (**B**) Quantification of the frequency of T cells as a percent of total CD45^+^ leukocytes in the PBMCs of individuals. (**C**) Quantification of the frequency of individual T cell subsets as a percent of CD3^+^ events. (**D**) Quantification of T cell subsets as a percent of CD4^+^ T cells. *p <* *0.05, **p <* *0.01, ***p <* *0.001, ****p <* *0.0001. DNT, double-negative T cell; Tfh, T follilcular helper cell; Treg, regulatory T cell.

To understand T cell development in newborn patients with lymphopenia, we applied 40+ plex single-cell mass cytometry (CyTOF) designed for immune-profiling PBMCs. T cell populations in both the twins as well as their father were distinct from healthy pediatric controls or adults when analyzed by t-SNE (Fig. 1A). Interestingly, the father’s T cell distribution was more similar to the healthy pediatric donors than the twins or healthy adult samples. Despite some improvement in T cell counts over time in patients with FOXN1 heterozygous mutations such as their father (Fig. 1B), the T cell compartment of those patients was not normalized compared with healthy adult donors. Indeed, although the father’s CD4^+^ and CD8^+^ T cell frequencies improved over time, the phenotype of his CD4^+^ T cells was significantly skewed to Th2, composing ∼50% of his total CD4^+^ T cells (Fig. 1C). Also notable were slightly elevated DNT frequencies in the twins, which may have been premature thymocytes that egressed from the thymus and/or failed to undergo thymic selection (Fig. 1D).

**FIGURE 2.**
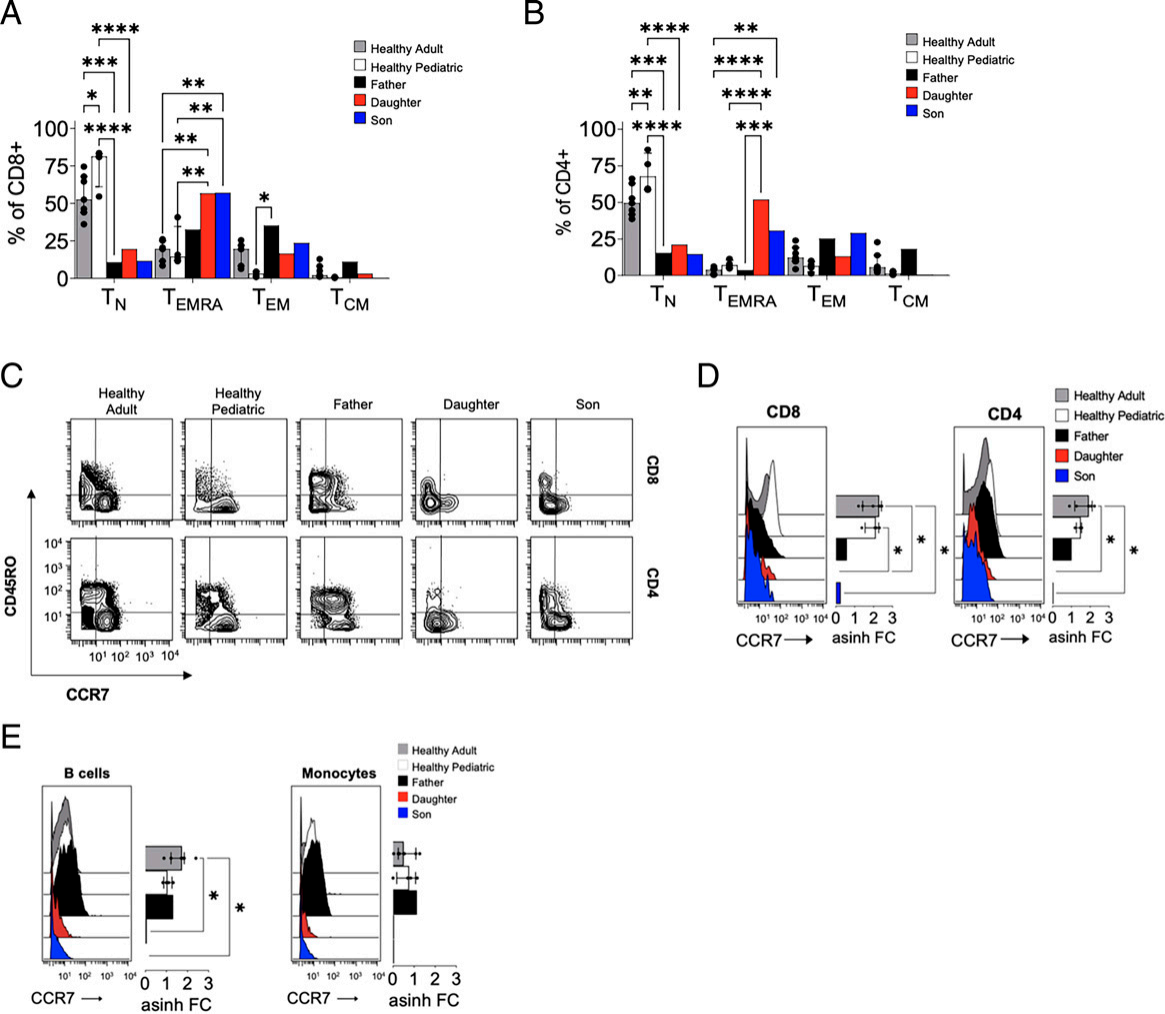
CCR7 dysregulation in FOXN1 patients. (**A**) Quantification of the frequency of CD8 memory T cell subsets as a percent of CD8^+^ T cells. (**B**) Quantification of the frequency of CD4 memory T cell subsets as a percent of CD4^+^ T cells. (**C**) Representative biaxial plots indicating the frequency of memory CD8 and CD4 T cell populations within each individual. (**D**) Representative histograms indicating the arcsinh-transformed median mass intensity of CCR7 (quantification of CCR7 median mass intensity). (**E**) Representative histograms indicating the arcsinh-transformed median mass intensity of CCR7 in CD19^+^ B cells and CD14^+^ monocytes. Quantification of CCR7 median mass intensity indicated by median expression ± interquartile range. *p <* *0.05, **p <* *0.01, ***p <* *0.001, ****p <* *0.0001.

Standard cell surface markers were used to classify memory populations of T cells. Both twins and their father demonstrated a significant depletion of naive CD4 and CD8^+^ T cells (Fig. 2A, 2B), whereas only the twins exhibited an expanded effector memory T cells re-expressing CD45RA (T_EMRA_) population. Consistent with an increase in T_EMRA_ cells was a decrease in naive T cells defined by CCR7 and CD45RO expression. However, one of these markers, CCR7, has been previously reported to be dysregulated on thymocytes by a heterozygous loss of *Foxn1* (17). Upon further analysis, what initially looked like an abundance of T_EMRA_ cells in the twins were instead naive T cells that failed to express CCR7 (Fig. 2C, 2D). Interestingly, the lack of CCR7 expression could also be seen in their B cell and monocyte populations (Fig. 2E). Taken together, these results reveal a failure of immune cells to drive expression of CCR7 in the presence of the c.1205del FOXN1variant.

### Expression of c.1205del FOXN1 variant protein

**FIGURE 3.**
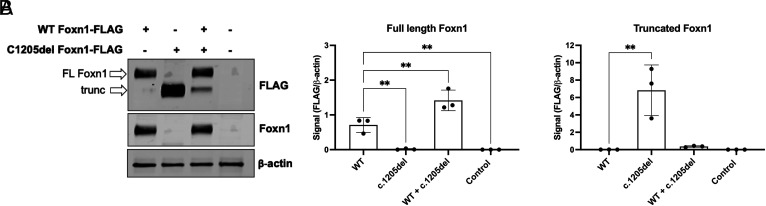
Truncated FOXN1 protein in c.1205del variant. (**A**) Representative immunoblot from 293T cells transfected with either FLAG-tagged WT Foxn1, c.1205del, both, or no DNA control. Protein expression was detected by anti-FLAG, anti-Foxn1, or anti–β actin Ab. (**B**) Quantification of the full-length Foxn1 protein or truncated protein from three independent experiments. Significance was determined by one-way ANOVA with a Sidak multiple comparisons test. **p <* *0.01.

To investigate potential pathogenic mechanisms of the c.1205del variant, we expressed FLAG-tagged WT or c.1205del FOXN1 proteins in cultured 293T cells. Interestingly, the c.1205del FOXN1 variant produced a truncated protein product that was detected by the anti-FLAG Ab, but not the anti-Foxn1 Ab that targets the C-terminal region that would be indicative of protein truncation (Fig. 3A). Despite expression driven by the same promoter and equal protein amounts loaded for immunoblot analysis, the c.1205del FOXN1 variant resulted in significantly more Foxn1 protein than did the WT plasmid. To recapitulate the heterozygous patient scenario, both WT and c.1205del FOXN1 were cotransfected. Both full-length and truncated Foxn1 were detected, but with an increased proportion of full-length and much reduced c.1205del FOXN1 (Fig. 3B). Of note, control cells that received neither plasmid had nondetectable levels of Foxn1 protein, indicating that there was no meaningful endogenous FOXN1 expression in the 293T cells for these experiments. Due to the varying protein levels between the WT and c.1205del FOXN1 variant, all subsequent experiments were performed using a 1:2 ratio of c.1205del to WT plasmid, allowing for comparisons with similar protein expression.

**FIGURE 4.**
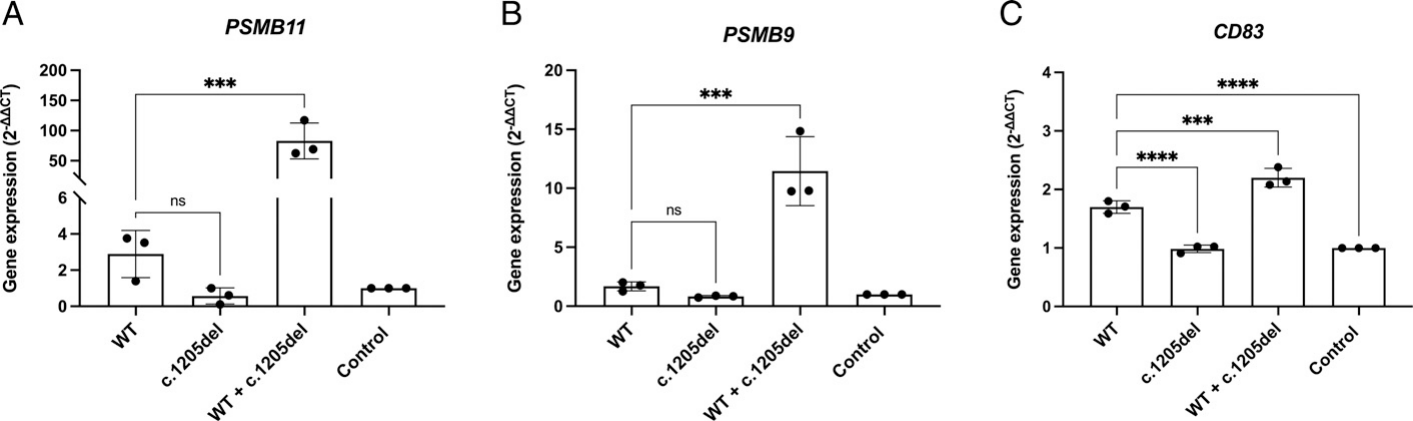
FOXN1 target genes have distinct responses to coexpression of WT and mutant FOXN1. 293T cells were transfected with either WT FOXN1, c.1205del FOXN1, or both. The control samples were not transfected with either plasmid and were used to calculate gene expression as fold change for each experiment. (**A**–**C**) Gene expression of PSMB11 (A), PSMB9 (B), and CD83 (C) was determined by quantitative RT-PCR and normalized to GAPDH expression. Results are from technical duplicates from three independent experiments. Significance was determined by one-way ANOVA with a Sidak multiple comparison test. ***p <* *0.001, ****p <* *0.0001. ns, not significant.

We next measured gene expression of validated FOXN1 target genes (17) to determine whether the c.1205del FOXN1 protein is able to activate their gene expression similar to WT FOXN1, and whether pathogenicity of this variant could be due to dominant negative effects on WT FOXN1. Although not statistically significant, there was a downward trend of c.1205del FOXN1 on the activation of both *PSMB9* (Fig. 4A) and *PSMB11* transcription (Fig. 4B) compared with WT. However, PSMB gene expression was only modestly induced in 293T cells with FOXN1 transfection. Surprisingly, coexpression of WT and c.1205del FOXN1 resulted in a significant increase in *PSMB9* and *PSMB11* transcripts (Fig. 4A, 4B). In the case of another FOXN1 target gene, *CD83*, the c.1205del variant failed to activate gene expression compared with the WT, but addition of WT was able to rescue gene expression when coexpressed (Fig. 4C). Therefore, in 293T cells, WT FOXN1 function could be enhanced to aberrant levels by the presence of the c.1205del variant for some gene targets (*PSMB*s), but WT FOXN1 rescued insufficient activity of the variant on other targets (*CD83*).

### DNA binding of FOXN1 variant to promoter binding sites

**FIGURE 5.**
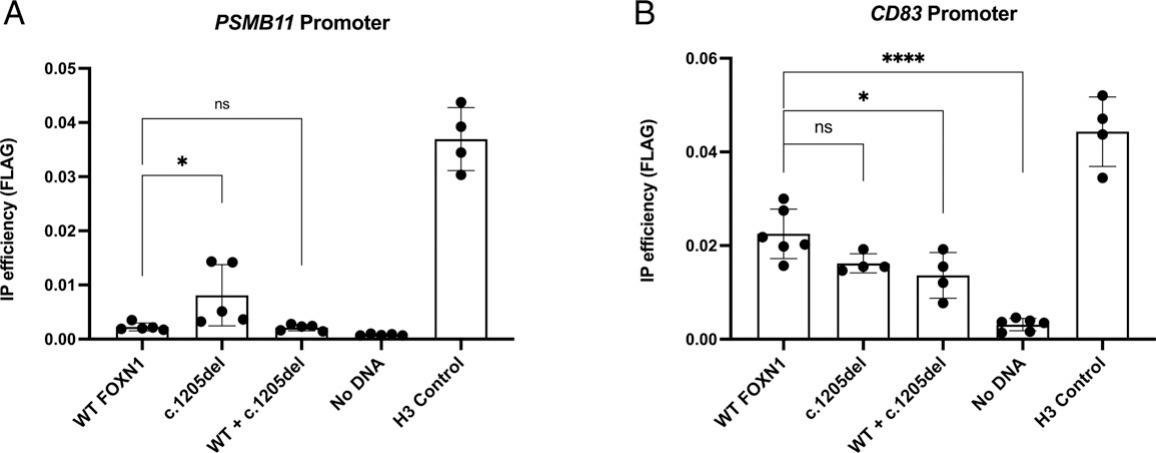
FOXN1 DNA binding to PSMB11 and CD83 promoters. (**A**) PSMB11 promoter. (**B**) CD83 promoter. All samples were immunoprecipitated with anti-FLAG Ab or an isotype control Ab. Immunoprecipitation efficiency was calculated by normalization of anti-FLAG sample above the isotype control signal. H3 control is a positive control where anti–histone H3 Ab was used for the immunoprecipitation. Significance was determined by one-way ANOVA with a Sidak multiple comparison test. *p <* *0.05, ****p <* *0.0001. ns, not significant.

Given the differential effects of the c.1205del variant on gene expression of PSMB9/11 and CD81, we asked whether this variant could bind to the FOXN1 binding motifs in their promoter regions. Transfected cells were subjected to ChIP with an anti-FLAG Ab and the resulting DNA was measured with primers designed to flank the FOXN1 binding motifs (GACGC) (17) in the PSMB11 and CD83 promoters (Fig. 5). Surprisingly, the c.1205del variant of FOXN1 was enriched for binding at the PSMB11 promoter compared with WT (Fig. 5A), despite promoting less gene expression in previous experiments (Fig. 4A). Therefore, the c.1205del variant does not demonstrate any defect in DNA binding, at least at the *PSMB11* promoter site. Interestingly, coexpression of both Foxn1 variants lost the enhanced binding efficiency seen by the c.1205del variant alone. Next, we examined DNA binding to the *CD83* promoter. In contrast to *PSMB11*, the c.1205del variant had a modest decrease in binding efficiency compared with WT FOXN1 (Fig. 5B). Furthermore, coexpression of both proteins revealed a dominant-negative effect of the c.1205del variant with WT FOXN1 to bind, with significantly less DNA binding than WT FOXN1 alone. However, the amount of FOXN1 bound to the *CD83* promoter may be sufficient to drive gene expression given the rescue of CD83 transcription seen by WT Foxn1 previously (Fig. 4C).

## Discussion

The T cell lymphopenia of *FOXN1* heterozygous patients has been well described (6, 7); however, more comprehensive immunophenotyping of twins along with their father allowed for further insight to a possible mechanism of resolution over time. We describe that despite homeostatic proliferation, adult patients with FOXN1 heterozygous mutations still do not have normalization of their T cell compartments. Even though T cell numbers normalized in their 30-y-old father, the distribution of memory subsets looked more like a pediatric healthy control sample than that of an adult of similar age.

We found that T cells from these heterozygous patients lack CCR7, uncovering a (to our knowledge) novel insight into the naive T cell populations that these patients do express. It is not clear whether this is due to a failure of thymocytes to express CCR7 ligands in the thymus, which then impacts downstream T cell selection and CCR7 upregulation in mature thymocytes. However, the B cells and myeloid cells also failed to express CCR7 in these patients, which would be independent of thymic selection or CCR7 ligands in the thymus. Nonetheless, we can speculate that the homing function of T cells and B cells is dysregulated without this receptor to guide them to lymph nodes. Ultimately, standard memory or cell surface markers may be altered in patients with poorly characterized immunodeficiencies or inborn errors of immunity, such as described in this study.

We used both human PBMC samples as well as cell line work to help further characterize this variant. For investigation into the mechanism, our main limitation is not having true TECs to model this variant. The cells we are using might have a completely distinct epigenetic environment, which makes some of the FOXN1 target genes inaccessible. Nonetheless, we were able to uncover some interesting findings that make it clear that some FOXN1 variants can be pathogenic by multiple mechanisms and cannot be explained by simple haploinsufficiency.

Further investigation is needed to determine whether this truncated protein product can bind and/or dimerize with WT FOXN1. Able to bind DNA, the variant may compete with WT FOXN1 for binding sites. In the example of the *PSMB11* gene, the variant bound more efficiently to the target site than did the WT. Next, we noted that there was a large discrepancy in the amount of protein detected in cells expressing WT FOXN1 or the variant; this may suggest a problem with protein turnover of this variant, which requires further testing. Furthermore, it is unclear how the truncated protein interacts with other chromatin binding factors that could impact the aggregation of FOXN1 that condensate in the nucleus (9). Lastly, we were able to further investigate a variant of FOXN1, which has since been characterized as pathogenic by the commercial genetic company used for testing. We recognize the impact that this translational work had on our patient, as ultimately the decision was made to monitor the T cell lymphopenia serially, without intervention. Utilizing a robust immunophenotyping platform allows clinicians and scientists to merge their work into better understanding and caring for patients.

## Supplementary Material

Supplemental Material (PDF)

## References

[1] Wang, H. X., W. Pan, L. Zheng, X. P. Zhong, L. Tan, Z. Liang, J. He, P. Feng, Y. Zhao, Y. R. Qiu. 2020. Thymic epithelial cells contribute to thymopoiesis and T cell development. Front. Immunol. 10: 3099. 32082299 10.3389/fimmu.2019.03099 PMC7005006

[2] Su, D. M., S. Navarre, W. J. Oh, B. G. Condie, N. R. Manley. 2003. A domain of Foxn1 required for crosstalk-dependent thymic epithelial cell differentiation. Nat. Immunol. 4: 1128–1135. 14528302 10.1038/ni983

[3] Romano, R., L. Palamaro, A. Fusco, G. Giardino, V. Gallo, L. Del Vecchio, C. Pignata. 2013. *FOXN1*: a master regulator gene of thymic epithelial development program. Front. Immunol. 4: 187. 23874334 10.3389/fimmu.2013.00187 PMC3709140

[4] Rota, I. A., F. Dhalla. 2017. FOXN1 deficient nude severe combined immunodeficiency. Orphanet. J. Rare. Dis. 12: 6. 28077132 10.1186/s13023-016-0557-1 PMC5225657

[5] Pignata, C., A. Fusco, S. Amorosi. 2009. Human clinical phenotype associated with FOXN1 mutations. Adv. Exp. Med. Biol. 665: 195–206. 20429426

[6] Bosticardo, M., Y. Yamazaki, J. Cowan, G. Giardino, C. Corsino, G. Scalia, R. Prencipe, M. Ruffner, D. A. Hill, I. Sakovich, . 2019. Heterozygous *FOXN1* variants cause low TRECs and severe T cell lymphopenia, revealing a crucial role of FOXN1 in supporting early thymopoiesis. Am. J. Hum. Genet. 105: 549–561. 31447097 10.1016/j.ajhg.2019.07.014 PMC6731368

[7] Du, Q., L. K. Huynh, F. Coskun, E. Molina, M. A. King, P. Raj, S. Khan, I. Dozmorov, C. M. Seroogy, C. A. Wysocki, . 2019. *FOXN1* compound heterozygous mutations cause selective thymic hypoplasia in humans. J. Clin. Invest. 129: 4724–4738. 31566583 10.1172/JCI127565 PMC6819092

[8] Chen, L., S. Xiao, N. R. Manley. 2009. Foxn1 is required to maintain the postnatal thymic microenvironment in a dosage-sensitive manner. Blood 113: 567–574. 18978204 10.1182/blood-2008-05-156265 PMC2628364

[9] Rota, I. A., A. E. Handel, S. Maio, F. Klein, F. Dhalla, M. E. Deadman, S. Cheuk, J. A. Newman, Y. S. Michaels, S. Zuklys, . 2021. FOXN1 forms higher-order nuclear condensates displaced by mutations causing immunodeficiency. Sci. Adv. 7: eabj9247. 34860543 10.1126/sciadv.abj9247 PMC8641933

[10] Roe, C. E., M. J. Hayes, S. M. Barone, J. M. Irish. 2020. Training novices in generation and analysis of high-dimensional human cell phospho-flow cytometry data. Curr. Protoc. Cytom. 93: e71. 32250555 10.1002/cpcy.71 PMC7682619

[11] Leelatian, N., D. B. Doxie, A. R. Greenplate, J. Sinnaeve, R. A. Ihrie, J. M. Irish. 2017. Preparing viable single cells from human tissue and tumors for cytomic analysis. Curr. Protoc. Mol. Biol. 118: 25C.1.1–25C.1.23. 10.1002/cpmb.37 PMC5518778 28369679

[12] Leelatian, N., K. E. Diggins, J. M. Irish. 2015. Characterizing phenotypes and signaling networks of single human cells by mass cytometry. Methods Mol. Biol. 1346: 99–113. 26542718 10.1007/978-1-4939-2987-0_8 PMC4656023

[13] Kaviany, S., T. Bartkowiak, D. E. Dulek, Y. W. Khan, M. J. Hayes, S. G. Schaefer, X. Ye, D. O. Dahunsi, J. A. Connelly, J. M. Irish, J. C. Rathmell. 2022. Systems immunology analyses of STAT1 gain-of-function immune phenotypes reveal heterogeneous response to IL-6 and broad immunometabolic roles for STAT1. Immunohorizons 6: 447–464. 35840326 10.4049/immunohorizons.2200041 PMC9623573

[14] Finck, R., E. F. Simonds, A. Jager, S. Krishnaswamy, K. Sachs, W. Fantl, D. Pe’er, G. P. Nolan, S. C. Bendall. 2013. Normalization of mass cytometry data with bead standards. Cytometry A 83: 483–494. 23512433 10.1002/cyto.a.22271 PMC3688049

[15] Katz, G., K. Voss, T. F. Yan, Y. C. Kim, R. L. Kortum, D. W. Scott, A. L. Snow. 2018. FOXP3 renders activated human regulatory T cells resistant to restimulation-induced cell death by suppressing SAP expression. Cell. Immunol. 327: 54–61. 29454648 10.1016/j.cellimm.2018.02.007 PMC5889721

[16] Newman, J. A., H. Aitkenhead, A. E. Gavard, I. A. Rota, A. E. Handel, G. A. Hollander, O. Gileadi. 2020. The crystal structure of human forkhead box N1 in complex with DNA reveals the structural basis for forkhead box family specificity. J. Biol. Chem. 295: 2948–2958. 31914405 10.1074/jbc.RA119.010365 PMC7062188

[17] Žuklys, S., A. Handel, S. Zhanybekova, F. Govani, M. Keller, S. Maio, C. E. Mayer, H. Y. Teh, K. Hafen, G. Gallone, . 2016. Foxn1 regulates key target genes essential for T cell development in postnatal thymic epithelial cells. Nat. Immunol. 17: 1206–1215. 27548434 10.1038/ni.3537 PMC5033077

