# Supplementary material for: Peripheral T Cell Development and Immunophenotyping of Twins with Heterozygous FOXN1 Mutations
Source: Immunohorizons. 2024 Jul 15;8(7):492–9. doi: 10.4049/immunohorizons.2400006 (PMC11294276; doi:10.4049/immunohorizons.2400006)
Supplement: Supplemental Material (PDF) [file IH_2400006_Supplemental_1.pdf]

Supplemental Materials: Early T cell Development and Immunophenotyping of Twins with Heterozygous *FOXN1* Mutations

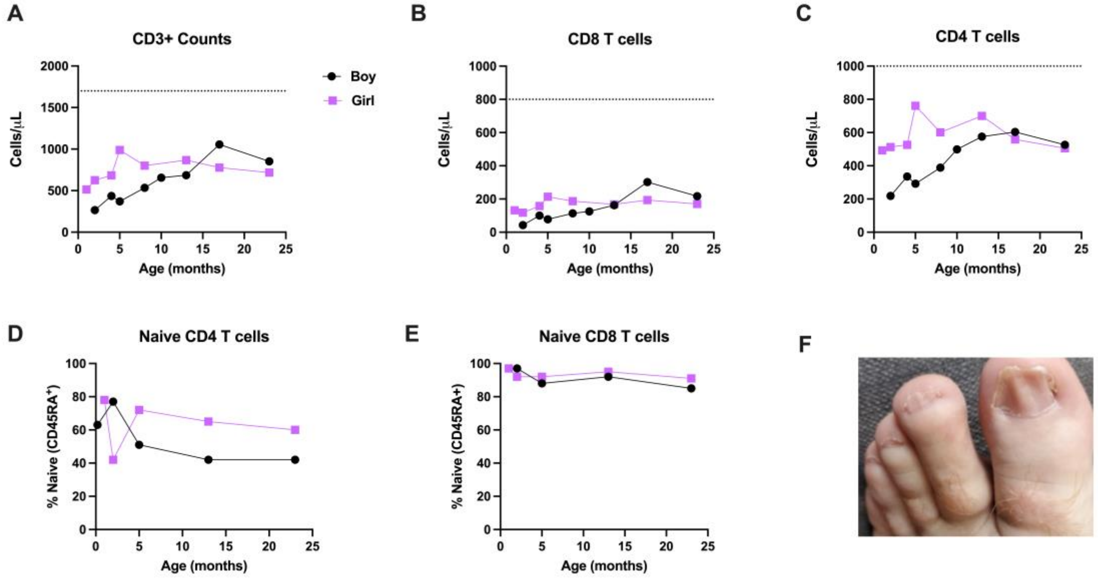

Supplemental Figure 1. T cell counts and nail dystrophy. Clinical testing for T cell counts performed on the twins for their first 23 months is shown. A) Total CD3+ counts, B) CD8 T cell counts, and C) CD4 T cell counts. The dotted line represents the lower limit of the normal range. (D+E) Percent of naïve CD4 and CD8 T cells over time. F) Toenail phenotype of the twins' Dad.
